# Phage-Phagocyte Interactions and Their Implications for Phage Application as Therapeutics

**DOI:** 10.3390/v9060150

**Published:** 2017-06-14

**Authors:** Ewa Jończyk-Matysiak, Beata Weber-Dąbrowska, Barbara Owczarek, Ryszard Międzybrodzki, Marzanna Łusiak-Szelachowska, Norbert Łodej, Andrzej Górski

**Affiliations:** 1 Bacteriophage Laboratory, Ludwik Hirszfeld Institute of Immunology and Experimental Therapy, Polish Academy of Sciences, Rudolfa Weigla Street 12, 53-114 Wroclaw, Poland; weber@iitd.pan.wroc.pl (B.W.-D.); owczarek@iitd.pan.wroc.pl (B.O.); mbrodzki@iitd.pan.wroc.pl (R.M.); marzena@iitd.pan.wroc.pl (M.Ł-.S.); norbert.lodej@iitd.pan.wroc.pl (N.Ł.); agorski@ikp.pl (A.G.); 2 Phage Therapy Unit, Ludwik Hirszfeld Institute of Immunology and Experimental Therapy, Polish Academy of Sciences, Rudolfa Weigla Street 12, 53-114 Wroclaw, Poland; 3 Department of Clinical Immunology, Transplantation Institute, Medical University of Warsaw, Nowogrodzka Street 59, 02-006 Warsaw, Poland

**Keywords:** phagocytes, phages, intracellular killing of bacteria, phagocytosis, monocytes, granulocytes, dendritic cells

## Abstract

Phagocytes are the main component of innate immunity. They remove pathogens and particles from organisms using their bactericidal tools in the form of both reactive oxygen species and degrading enzymes—contained in granules—that are potentially toxic proteins. Therefore, it is important to investigate the possible interactions between phages and immune cells and avoid any phage side effects on them. Recent progress in knowledge concerning the influence of phages on phagocytes is also important as such interactions may shape the immune response. In this review we have summarized the current knowledge on phage interactions with phagocytes described so far and their potential implications for phage therapy. The data suggesting that phage do not downregulate important phagocyte functions are especially relevant for the concept of phage therapy.

## 1. Introduction

Phagocytes are the main component of human innate immunity. In the human body phagocytic cells are neutrophils, monocytes, tissue macrophages and dendritic cells [1]. Their function is based on the non-specific defense of the body against pathogens and substances produced by them, e.g., toxins, which involves uptake of the antigen and its degradation in the phagosome, where degrading enzymes are present [2,3]. After the pathogens’ entry into the body the number of circulating leukocytes increases. Leukocytosis is observed in the course of infection or inflammation [4].

Mature neutrophils are short-lived cells, but they circulate in a huge amount (their production is about 10^9^ cells/kg/day; they represent 50–70% of all leukocytes in an adult’s peripheral blood [4,5]. They digest extracellular pathogens and their toxins. Neutrophils achieve high effectiveness in killing bacteria by combining the action of reactive oxygen species (ROS) and substances contained in the granules of these cells [6].

Monocytes from the bone marrow enter the peripheral blood, where they circulate for 2–3 days [7], before passing into the tissues and converting into macrophages—present in the connective tissue (histiocytes), liver (Kupffer cells), spleen, lungs (alveolar macrophages) and central nervous system (microglial cells) [8]. The percentage of monocytes does not exceed 10% of the total number of white blood cells. Their role is based on removing potentially harmful particles, e.g., microorganisms, dead cells, or their residues, processing them and presenting antigens to lymphocytes [1]. This is one of many mechanisms linking the innate to adaptive immunity. Monocytes also regulate the function of antigen presenting cells, e.g., dendritic cells. The main tasks of macrophages include removal of pathogens or particles potentially harmful in the process of phagocytosis, antigen presentation to T cells, and the production of cytokines [8]. Splenic macrophages are important especially in the removal of capsular bacteria (e.g., *Neisseria meningitidis*). Macrophages have a weaker respiratory burst, and their mature forms neither show the presence of myeloperoxidase nor include numerous antimicrobial proteins. The cytoplasm of macrophages does not possess granules with NADPH oxidase, which is compensated by the induction of cytokine production. Together, bacteria, their products, inflammatory cytokines, and interferon stimulate NO synthase, which is the main path of bactericidal activity of macrophages [9].

Dendritic cells (DC) derive from myeloid progenitor cells in the bone marrow, and they colonize tissues. One of these cell subtypes—myeloid dendritic cells—is functionally and developmentally related to macrophages. They have a common precursor with macrophages, and may also transform into macrophages [10]. Immature forms of dendritic cells are able to undergo phagocytosis, whereas upon activation they migrate to the lymph nodes, where the cells present antigens to T lymphocytes.

Phagocytosis leads to the destruction of pathogens penetrating the body [4]. It occurs as a result of direct or indirect (e.g., due to opsonization) identification of microorganisms [11]. The process consists of: targeted traffic of phagocytes (chemotaxis) toward the source of infection; recognition of foreign particles by membrane receptors located on the surface of phagocyte e.g., lectins; absorption of the particles into the vacuole (phagosome); degranulation and release of intracellular granules’ enzymes into the phagosome (phagolysosome formation); the production of ROS by the enzyme complex which is located in the cell membrane and/or reactive forms of nitrogen; and killing and digesting the material in the phagosome.

Bacteriophages are the most numerous structures in the biosphere [12,13,14]. Because they are so abundant, they constantly interact with organisms, and not only with bacteria that they infect. They are present in the human microbiome, and therefore they are well tolerated when used in phage therapy [15,16]. It has been proved that they can adhere to the eukaryotic cells [17]. Moreover, phage therapy increases the exposure of human (the patient’s) cells to these prokaryotic viruses, and it may exert an effect especially on immune cells. Nowadays, phages are seen as innovative components as a hope in fighting the antibiotic resistance [18], and this form of therapy is becoming increasingly popular and more frequently used because of antibiotics’ crisis [19]. Therefore, it is extremely significant from the safety point of view to determine if phages do not impair the functions of phagocytes, even if they are applied in immunocompromised patients, who constitute a significant proportion of patients who qualify for experimental phage therapy [20].

Eight years ago, in their paper, Kurzępa et al. (2009) stated that “the present knowledge about phage interactions with cellular components of the mammalian immune system is sparse and insufficient, especially considering the increasing interest in the application of these viruses in human life. We believe that continuation of such research is indispensable” [21]. In subsequent years new and important data have accumulated which shed more light on phage-phagocyte interactions.

## 2. Phagocytosis of Bacteriophages

The process of bacteriophage phagocytosis by phagocytic cells has been described. Although phages are bacterial viruses, interactions between them and human or animal immune cells have been observed.

As demonstrated by Aronov et al. (1964), bacteriophages can be ingested by phagocytic cells during phagocytosis [22]. Phagocytosis of the *Escherichia coli* T2 bacteriophage by macrophages and rabbit peritoneal neutrophils as well as disintegration of phage virions in vitro was described. The inactivation of phages localized within macrophages was observed 2 h after phagocytosis. Phages are capable of attaching to the cell membrane of leukocytes [23]. It was suggested that also phage vectors enter mammalian cells by endocytosis mediated by integrins. Following their entry into cells in the phagocytosis-like process, the vectors initially retained their lytic activity [24]. Then, they were transported to lysosomes, where the phages were inactivated in the acidic environment in the presence of 100 µM of chloroquine.

Barfoot et al. (1989) observed phagocytosis of the T4 phage by dendritic cells in vitro [25]. This process was more effective in the case of the T4 phage and the influenza virus particles than the synthetic particles (latex). Active virions were more effectively phagocytosed. What is more, phages were localized in phagolysosomes. During phagocytosis the phage’s outer coat was removed. Recently, Kazmierczak et al. (2014) obtained T4 phage particles (containing the fusion protein GFP + Hoc) showing green fluorescence [26]. By incubating them with the murine macrophage cell line J774A.1 green fluorescence was observed within macrophages. These observations confirmed that these cells have the ability to phagocytose the T4 phage. Also Hodyra-Stefaniak et al. (2015) visualized phage degradation by splenocytes and identified the phage particle within macrophage compartments (using super-resolution structural-illumination microscopy and green fluorescent protein-labeled phage) [27]. The results can contribute to further innovative development and use of phages for phage therapy, e.g., for the treatment of infections caused by intracellular bacteria or monitoring the location of the phages in living organisms or tissues. These above findings confirm that phages may interact with phagocytic cells as well as penetrate them. Phage particles’ degradation within phagocytes may be observed, and may be one of the reasons for phage insusceptibility. However, whether particular phage particles would be degraded may be a phage’s individual feature and may determine the fate of the treatment using phage therapy.

## 3. Phage Influence on Phagocytosis of Bacteria

An interesting area seems to be research concerning whether phages may influence phagocytosis of bacteria. The first observations on the interactions of phages and immune cells were made in vivo and in vitro in the 1920s [28]. D ‘Herelle studied the effect of phages active against *Shigella* on phagocytosis of bacteria by guinea pig leukocytes. He observed that the index of phagocytosis of *Shigella* grew after 10 min’ incubation of bacteria, phages and leukocytes, compared to controls, in which the cells were incubated only with the bacteria in the absence of phage. This study revealed that bacteria that have acquired resistance to these specific phages also become resistant to phagocytosis. It suggested that this phenomenon could be explained by the fact that phages act as opsonins that facilitate phagocytosis of bacteria. Similarly, Nelson (1928) demonstrated in vivo that phagocytosis is a process which may be affected by phages active against S*taphylococcus aureus* [29]. Intravenous administration of phages resulted in an immediate increase in the ability of leukocytes to phagocytose bacteria susceptible to phage. But in the case of phage-resistant bacteria the strength of phagocytosis remained unchanged.

However, Młynarczyk et al. (1988) reported that lysogenic conversion may have an influence on the susceptibility of *S. aureus* to phagocytosis [30]. Intracellular killing (IK) of the lysogenic 8325-4 strain of *S. aureus* by granulocytes was weaker compared to the strain without integrated prophage. These results could be related to the fact that genes of some prophages may have an influence on the synthesis of the antiphagocytic surface receptors or may result from the presence of R plasmids in the *S. aureus* cells. Similarly, Secor et al. (2017) demonstrated that bone marrow dendritic cells internalized the *Pseudomonas aeruginosa* strain with integrated phage less than the bacterial strain itself. The bacterial strain producing the filamentous Pf4 phage was observed to be less susceptible to phagocytosis by macrophages than those not producing the Pf phage [31]. This is contradictory to Smith’s (1928) observations, which suggested that exposure of live bacteria to specific phage increases susceptibility to bacterial phagocytosis [32].

The effect of phage therapy on *S. aureus* phagocytosis by neutrophils of patients who were treated with phages was investigated [33]. The decrease in bactericidal activity of the phagocytes was observed in the case of patients whose phagocytes had shown impairment in phagocytosis already prior to the therapy. However, there was no correlation between changes in phagocytosis and the course of therapy. Interestingly, three months after the treatment with phages neutrophils of these patients started to gradually recover their capacity for phagocytosis. However, during the first weeks of phage therapy increased phagocytosis by neutrophils was observed in patients in whom the therapy gave good results, which would indicate that assessing phagocytosis may have a prognostic value in phage therapy [20].

Data presented herein show that there is no universal type of phage influence on phagocytosis either in vitro or in vivo. The observed effects may depend on, e.g., the type of phage, bacteria as well as type of phagocytes.

## 4. Phage Influence on Intracellular Killing of Bacteria

Intracellular killing of bacteria was shown to be influenced by phages. This process is fundamental for pathogens’ elimination by innate immunity. It was demonstrated that peripheral blood phagocytes’ (both polymorphonuclear leukocytes (PMNs) and peripheral blood mononuclear cells (PBMCs)) ability to kill bacteria (pathogenic and nonpathogenic) intracellularly, in patients suffering chronic infections who were qualified for experimental phage therapy, was impaired [34] More importantly, it was also found that phage therapy did not cause a further decrease in patients’ phagocytes’ ability to kill bacteria (both the pathogenic and the reference bacteria). What is more, in patients with urinary tract infections phage treatment corrected the weakened IK of nonpathogenic bacteria by monocytes. The results confirm the data derived from the studies conducted by Kurzępa-Skaradzińska et al. (2013) in vitro [35]. The authors reported that the phage preparations (both in lysate and purified form) did not affect the capacity of human phagocytes for IK of bacteria, regardless of the titer of the phage preparation and its specificity for the bacterial strain (homologous and heterologous). Moreover, our unpublished data obtained from mice with an experimentally induced urinary tract infection also indicated that the bacterial infection reduced the bactericidal capacity of phagocytes isolated from mouse spleen, regardless of the uropathogenic strain used to induce the acute urinary tract infection (*Enterococcus faecalis* or *P. aeruginosa*). The use of three consecutive doses of phage lysate after induction of the acute infection exerted a stimulatory effect on intracellular killing of bacteria by mouse splenocytes. This process was normalized to the level observed in the control group (phagocytes of healthy animals) 6 days after the infection induction. Our results confirm that phage therapy has no harmful effects on the bactericidal properties of peripheral blood phagocytes of patients treated with phage lysates.

The reduced ability of whole blood to kill methicillin-resistant *S. aureus* intracellularly was observed in BALB/c mice with experimentally induced diabetes compared to the bactericidal properties of blood of healthy animals (correlated with the glucose level in the blood) [36]. The combined use of linezolid and MR-10 phage active against *S. aureus* increased the bactericidal activity of mouse whole blood to kill bacteria intracellularly. What is more, our unpublished data (Jończyk-Matysiak et al.) also demonstrated that preincubation of the purified T4 phage with human peripheral blood phagocytes—PMNs or mononuclear cells—significantly increased the percentage of killed bacteria in vitro, compared to bacteria incubated only with the phagocytes. Similar effect of the PA1Ø phage (active against *P. aeruginosa*) and mouse neutrophils isolated from peripheral blood was observed against *P. aeruginosa* in vitro [37]. In the samples which contained both the phage and neutrophils, there was a significant decrease in bacterial titer, compared to samples containing the phage only, which suggested that the phage-neutrophil co-work may be essential for the efficient killing of bacteria in the mice. These findings indicate that phages may act synergistically with phagocytes in eliminating bacteria.

## 5. Phage Participation in the Killing of Intracellular Bacteria

Pathogens avoiding phagocytosis are a major clinical problem as they can remain alive inside the phagocytes [38]. The treatment of such infections may be inefficient because many antibiotics of proven efficacy in vitro against intracellular pathogens have little or no activity in vivo. In consequence, antibiotics are used in too low concentrations to be able to destroy pathogens or stimulate the phagocytes to carry out intracellular killing of these bacteria. Moreover, not all antibiotics of proven capacity to penetrate into the interior of phagocytes can destroy bacteria. Therefore, using phages that can penetrate phagocytes is an interesting and promising option.

According to Broxmeyer et al. (2002), lytic TM4 mycobacteriophage did not show the ability of intracellular lysis of mycobacteria, because it could not penetrate to the interior of macrophages [39]. Only phages supplied to a macrophage using a non-pathogenic strain of *Mycobacterium smegmatis* destroyed intracellular bacteria. While testing the effect of D29 mycobacteriophage on intracellular bacteria in vitro, the authors observed that the phage was able to kill the intracellular bacteria because it had penetrated inside the macrophages. Most likely, the difference in phages’ ability to penetrate to the interior of the tested phagocytes resulted from the difference in the phages’ structure; probably the TM4 phage had no molecules to enable it to adsorb to the macrophage membrane and penetrate to its interior.

Interestingly, Capparelli et al. (2007) observed that phage M^Sa^ active against *S. aureus* (including methicillin-resistant *S. aureus*) destroyed bacteria inside murine macrophages both in vitro and in vivo [40]. The phage alone could not penetrate into the interior of murine macrophages, whereas when supplied to the interior of the cells using *S. aureus* (after pre-incubation of the phage with *S. aureus* A170S strain), it efficiently killed bacteria that were localized intracellularly (approximately 70%). Interestingly, the MR-5 phage specific to *S. aureus* (which was adsorbed to engulfed bacterial cells), penetrated into murine peritoneal macrophages and caused a significant reduction in titer (a decrease of 2.5 log in 2 h) of *S. aureus* that was localized intracellularly [41]. Phage particles which were transferred to the inside of macrophages also significantly reduced the damage caused due to cytotoxic effects of bacteria on phagocytes. What is more, the killing was higher at a lower bacteriophage/bacteria ratio, and these findings correspond with observations of Baughn and Bonventre (1975), who reported that killing of *S. aureus* by mouse peritoneal macrophages was also higher when a lower multiplicity of infection (MOI) was used in the experiments [42].

These results could suggest a potential use of phages to treat such dangerous infections as those both local and systemic caused by intracellular pathogens.

## 6. Phages and Respiratory Burst

ROS are a strong “weapon” used by phagocytic cells to eliminate pathogens absorbed by phagocytosis. There are data concerning the effect of phages on the production of ROS which are produced by phagocytes after uptake of antigen [43,44,45]. The purified T4 phage only weakly induced the respiratory burst in monocytes and neutrophils, as compared to bacteria [43]. However, the inhibited production of ROS by neutrophils stimulated by bacterial antigens, such as lipopolysaccharide (LPS), it was observed, either when phages specific or non-specific to bacteria were used [43]. The ability of the T4 phage and its proteins to generate ROS in neutrophils and mononuclear cells from human blood was also tested, and these results pointed to a lack of proinflammatory action of both phage and their highly purified head proteins exposed on the phage surface: the major capsid protein (gp23), gp24, highly immunogenic outer capsid protein (Hoc), and small outer capsid protein (Soc) [45]. Similarly, Borysowski et al. did not show in vitro that both purified preparations and A3/r phage lysates (active against *S. aureus*) induced the respiratory burst in either neutrophils or monocytes [46].

The above findings are contradictory but most of them showed that phages do not stimulate ROS production and as a consequence they do not contribute to damage and destruction of uninfected tissues. The use of phage preparations for systemic infections (e.g., sepsis, during which the production of ROS may result in damage to tissues and organs) could reduce the harmful effects of degradation products of bacteria on tissues and organs of patients.

## 7. Phage Influence on the Other Steps of Phagocytosis

Despite phage ability to influence the uptake of bacteria and/or their killing intarcellularly with the use of ROS, there are data showing that phages may interact with phagocytes at different steps of phagocytosis, e.g., their recruitment or release of substances with bactericidal activity.

Secor et al. (2017) reported that the filamentous Pf4 bacteriophage active against *Pseudomonas aeruginosa* in vivo (murine pneumonia model) reduced both neutrophil recruitment and the production of cytokines [31]. The authors hypothesized about both the role of the phage in the development of chronic infection and evasion of the defense mechanism of the host. They stated that the production of the filamentous phage may cause a reduction in the dissemination and inflammation caused by increased adhesion of bacterial cells to the mucin. Moreover, the production of the Pf4 phage was probably associated with macrophage polarization, and phage-associated bacteria caused the expression of higher levels of M1 and M2 markers compared to the bacterial strain itself.

Also, the effects of the A3/R purified phage and its lysate on degranulation of neutrophils’ primary and secondary vesicles was tested [47]. Blood samples were investigated by flow cytometry and the expression of markers of exocytosis–CD63 (primary granules) and CD66b (secondary granules)—was investigated. The researchers found that neither the A3R purified preparation nor the phage lysate did not induce degranulation in neutrophils. This result suggest that phage therapy should not induce significant phagocyte degranulation therefore confirming its safety and low incidence of side effects.

## 8. Phages and Inflammation

We have noted a marked anti-inflammatory effect of therapy in patients receiving phage treatment; in some of them the response was dramatic even though bacterial eradication had not been achieved. This suggested that phage can mediate their anti-inflammatory action by at least two mechanisms: eradicating bacterial infection and directly downregulating the activity of cells engaged in proinflammatory processes [48].

It was demonstrated that both the T4 bacteriophage and its head proteins: gp23, gp24, Hoc, and Soc did not stimulate the inflammatory mediators and inflammation-mediated factors, particularly pro-inflammatory cytokines [45]. The authors tested the cytokine profile both in vivo (mouse model, intraperitoneal injection) and in vitro (on cells isolated from human blood). When mouse bone marrow-derived dendritic cells were treated with the T4 phage and its capsid proteins, no significant production of cytokines such as IL-1α, IL-6, IL-12 and TNF-α was observed. Moreover, the authors investigated the effect of the T4 phage and its head proteins on the differentiation and activation of bone marrow-derived dendritic cells. They observed no changes in the expression profile of cell surface antigens, such as major histocompatibility complex (MHC) class II, CD40, CD86 and CD80. The obtained data support the safety of phage preparations’ application in medicine.

The effect of the T4 and A3/R purified phages and their lysates on differentiation of human myeloid dendritic cells has been investigated to some degree [49]. Neither of the tested phages influenced the expression of the markers responsible for maturation of DC and activation of T cells, such as CD40, CD80, CD83, CD86, CD1c, CD11c, MHC class II, PD-L1, PD-L2, TLR2, TLR4, and CCR7. It was also found that the phages did not influence the percentage of CD64 and DEC205 positive cells, which are known to be involved in phagocytosis. However, the T4 lysate significantly reduced the percentage of the CD64 and DEC205 positive cells, while the lysate significantly increased the percentage of the TLR4 and PDL-2 positive cells (in comparison with the control). Whereas, according to Górski et al. (2012) the T4 phage did not affect the percentage of TLR2 and TLR4^++^ cells, as was observed in in vitro experiments in both monocytes stimulated and those not stimulated by LPS [20]. Also in myeloid DC, which were differentiated with the addition of the A3/R lysate, the presence of DEC205 was reduced [49]. It was observed that the level of IL-12 produced by the myeloid DC in the presence of the T4 and A3/R phages was undetectable, which may confirm that phages do not have an influence on the activation of mDC. The authors suggested that the observed changes in DC marker expression may be caused by the components of bacterial cells contained in phage lysate. Also, Bocian et al. (2016) found that both the T4 purified preparation and its lysate significantly increased the percentage of monocytes with an activated phenotype (CD14^+^CD16^−^CD40^+^ and CD14^+^CD16^−^CD80^+^) in unactivated PBMCs while it had no effect on LPS-activated monocytes [50]. The authors stated that their observation may be important because it suggests that in patients infected with Gram-negative bacteria neither of the phage preparations applied in the therapy—purified phage or products of bacterial lysis in lysate form—stimulated monocytes. Ann et al. (2014) reported that the ES2 *Cronobacter sakazakii* phage activated dendritic cell maturation and caused an increase in the expression IL-12-p40 [51]. These findings may indicate that this phage can induce an inflammatory response.

Miernikiewicz et al. (2016) also showed that the short tail fiber protein (gp12) from the T4 phage, which mediates adsorption of the phage to *E. coli*, may bind and form complexes with LPS—one of the known pathogen-associated molecular patterns (PAMP) recognized by toll-like receptors [52]. The authors suggested that the phage protein may be a modulator of LPS-induced inflammation, because their results indicated that LPS in combination with gp12 did not cause any negative influence on mammalian cell proliferation (fibroblasts, endothelium), and it caused a decrease in the inflammatory response to LPS in a murine model (a reduction of the level of IL-1α and Il-6 in LPS-challenged mice). What is more, the gp12 protein itself had no proinflammatory activity in mice. The application of the protein to mice with inflammation induced by LPS caused a significant decrease in the infiltration of leukocytes into the liver and spleen. Also, no cytotoxic effect on the growth and viability of tested cell lines, either animal (murine fibroblasts, Balb3T3) or human (HSkMEC), was observed when the gp12 protein was applied, nor were there any harmful effects on tissues of mice treated only with the protein. What is more, the authors suggested the possibility to use the tested protein as a potential anti-inflammatory drug.

The response to *Pseudomonas* F8 and T4 phages has been described based on the mouse model [27]. The authors found that in animals with induced systemic inflammatory response syndrome (SIR) with LPS the concentration of phages in the spleen was significantly lower (2.58 log) than in the case of the control group, which the authors associated with the decrease in phage particles circulating in the blood of mice with inflammation 1 hour after phage administration. The obtained results may be explained by the fact that spleen phagocytes isolated from SIR mice could phagocytose phages more effectively compared to control mice.

A promising perspective would be an idea of using the combined action of phages and monocytes and their properties Bacteriophages are characterized by a total negative electric charge on the surface (the negative zeta potential of −30 to −10 mV) [53,54,55,56,57]. Such a physical property could provide a basis for using the modified phage particles to bind to the so-called inflammatory monocytes expressing chemokine receptor type 2, CCR2, which is responsible for the recruitment of these cells to sites of inflammation. The incorporation of phage particles to monocytes (by positively charged scavenger receptors on the surface of these cells) could direct these cells to the spleen, where they are subjected to apoptosis, as was observed in the case of using negatively charged biodegradable microparticles [58]. This could be a breakthrough in the treatment of pathologies caused by the so-called inflammatory monocytes.

The presented ability of phages tested so far not to participate in the development of inflammation as well as their suggested anti-inflammatory action may allow them to be an important tool for application in the treatment of infections and inflammation.

## 9. Indirect Interaction with Immune Cells

The effect of phages’ interactions with the immune cells may be indirect, e.g., by the components of phage lysate that contains bacterial residues. Phage lysates may modulate the immune system. Zimecki et al. (2003) demonstrated that purified preparations of A20/R phage active against *S. aureus* have a costimulatory effect on murine splenocytes activated by suboptimal concentration of concanavalin A [59]. The results indicate that the therapeutic effect of phage preparations may be related not only to the phage bactericidal activity, but also to the stimulation of the cells of the immune system (e.g., cytokine production) and increased ability to proliferate under the action of mitogens. Stapels et al. (2014) suggested that *S. aureus* strains have the ability to synthesize serine proteinase inhibitors, which could be important in the inhibition of inflammation [60]. This could suggest a potential mechanism for the anti-inflammatory effect of staphylococcal phage lysates. Strengthening immunity can be an effect of the products presented in the phage preparations, e.g., in staphylococcal phage lysates [20].

As suggested by Górski et al. (2012), the therapeutic effect of phage preparations may be connected not only with the elimination of bacteria, but may also depend on the normalization of inflammatory markers associated with bacterial infection [20]. A decrease in C-reactive protein (CRP) levels was observed in patients treated with phage therapy [61]. Phage therapy (phage lysates) did not show proinflammatory action in patients [17]. An increase of leukocytosis and erythrocyte sedimentation rate (ESR) was not observed. Moreover, phage therapy accelerated the circulation of neutrophils, which confirmed a significant increase in the level of immature forms of neutrophils in the blood with a simultaneous decrease in mature cells [33]. However, our previous observations conducted on patients (*n* = 51) treated with phage therapy showed that the therapy does not appear to have a significant impact on the percentage of the different fractions of circulating blood leukocytes, so it probably does not affect the granulopoiesis or myelopoiesis [62]. What is more, there was no significant influence of phage therapy on inflammatory markers, either ESR or CRP, in patients who received phage therapy, and—at least in some patients—phage therapy may cause lowering of those markers which may suggest that experimental phage therapy does not stimulate the inflammation process. The results may be of practical importance as they confirm the safe use of phage lysates in patients treated with phage therapy, especially in patients with congenital and acquired immune deficiencies.

## 10. Practical Implications of Phage-Phagocyte Interactions

Pathogenic viruses’ interactions with cells of the immune system include binding of virions to cells, phagocytosis and associated mechanisms (e.g., the production of ROS) and apoptosis. There exists no uniform response of the phagocytic cells to a pathogenic virus. Viral infection results in the synthesis of interferon, and its release to surrounding tissue leading to the appearance of virus-specific receptors on the surface of adjacent not-yet-infected host cells [63]. Viral particles can be degraded by neutrophil-associated proteins (contained in the granules), such as defensins, cationic proteins, and reactive oxygen species [64,65,66]. Activation of phagocytes caused by a viral infection is associated with the production of reactive oxygen species by the cells, as well as the release of cytokines, such as TNF and IL-1 [67,68]. Interleukin-1 stimulates the release of lactoferrin from neutrophils. This is important because of the ability to eliminate both the pathogen and the infected cells. Viruses (e.g., Sendai virus, influenza virus, cytomegalovirus, HIV) may contribute to increased production of ROS by phagocytes (murine splenocytes, human neutrophils, monocytes) [69,70,71]. Also, inhibition of apoptosis resulted in the accumulation of over-reactive neutrophils in tissues that can cause tissular damage, resulting in inflammation (e.g., neutrophils infected with HCMV) [72]. The aim of phagocytes in case of eukaryotic pathogenic virus is to destroy and remove with the use of all available mechanisms. Viral infection causes changes in the functioning of the immune system of the body in which the infection develops. Table 1 summarizes the comparison of the influence of pathogenic eukaryotic and nonpathogenic (phages) viruses on phagocyte functions. Based on the above information, the pathogenic viruses have a different mechanism of action on phagocytic cells of the immune system than bacterial viruses.

**Figure 1:**
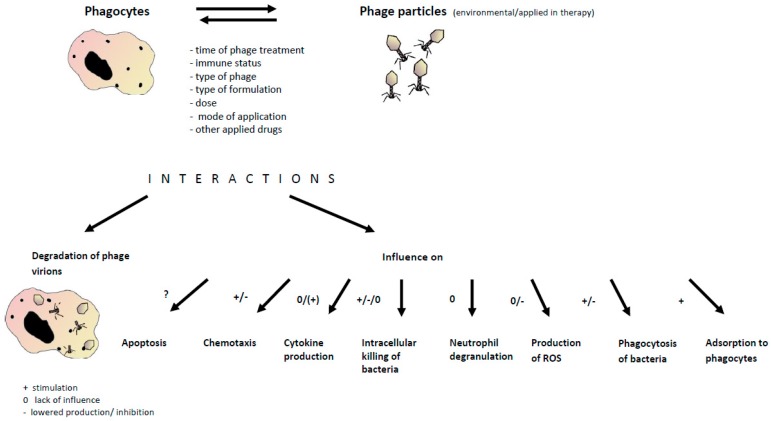
Summary of interactions between phages and phagocytes and possible implications for phage application in therapy. These interactions may be mediated by phages present in our environment and administered during phage therapy, and depend on a variety of factors (e.g., time of phage treatment, etc., as shown). As a result, various phagocyte functions may be affected which can have practical implications for the effectiveness of phage therapy.

Bacteriophages are not pathogenic for human and animals and although that they can interact with phagocytic cells and be adsorbed to the mammalian cells surface receptors, phagocytosed and degraded, they are not potentially threatening. Despite the fact that phages constantly interact with human organisms (they are present in drinking water, air, food, and soil), and they are a natural component of the biosphere, the therapeutic doses of phage particles contained in phage preparations are higher than those of environmental exposure [17]. Figure 1 presents summarized interactions between phages and phagocytes and possible implications for phage application in the therapy.

The data presented herein indicate that in the response to the phage the immune system does not produce cytokines and ROS and does not cause neutrophil degranulation. It indicates that the phage does not cause an inflammatory response that may be harmful for tissues. What is more, there is a possibility to stimulate the uptake of pathogenic bacteria which may help in controlling the infection not only by their antibacterial activity but as an effect of interactions with phagocytes. Therefore, it is of particular importance that the data presented herein indicated phages not to be toxic, unsafe or to cause harmful side-effects on immune cells in patients treated with phages. Because of phage variety more studies are needed, and especially those used in phage therapy should be evaluated.

## 11. Conclusions

Studies involving interactions with phages of immune cells are essential for the understanding of the role of phages in nature and rational use of phage therapy [20]. It is necessary to assess whether the phages contained in the formulations applied to the patients can stimulate the immune system to eliminate pathogenic bacteria, or decrease the antimicrobial activity of the immune system. The impact of phages on cells of the immune system is an interesting area for future research. Knowledge about the interaction between phages and mammalian cells, in particular phagocytes, is limited so far. Interactions between phages and mammalian cells of the immune system are based primarily on the immunogenic properties of phages (the production of specific antibodies by phage antigens) and immunomodulatory effects on the immune system of mammals (the function of phagocytes—such as phagocytosis, production of reactive oxygen species or proliferation of T lymphocytes). Studies on the effects of phages on immune cells may provide important information on the safety of phage therapy. As has been shown, phage-phagocyte interactions differ from interactions of phagocytes with pathogenic viruses.

Most studies have been conducted in vitro or in animal models, and more detailed studies especially in humans are needed. Moreover, there are still processes for which the influence of phages has not been described yet (e.g., apoptosis).

We believe that the recent progress in knowledge concerning the influence of phages on phagocytes and their interactions will significantly expand our knowledge about the role of phages and improve phages’ therapeutic applications.

## Figures and Tables

**Table 1:** The influence of pathogenic and nonpathogenic (phages) viruses on phagocyte functions.

Mechanism	Pathogenic Eukaryotic Viruses	Bacteriophages
**Cytokine production**	Epstein-Barr virus causes an increase in production of e.g., TNF-1α, IL-6, IL-8, IL-10 [73].	Lack of stimulation: IL-1α, IL-1β, IL-2, IL-6, IL- 10, IL-12, p40/p70, INT-γ, TNF-α (T4 phage and proteins on the surface of the phage head: gp23, gp24, Hoc, Soc) [45]. Inhibition of production of NFκB by human mononuclear cells induced by human herpesvirus-1 [74].
**Chemotaxis of phagocytes**	JEV stimulates production of the factor stimulating neutrophil chemotaxis by murine macrophages [75] influenza virus significantly decreased neutrophils migration activity [71].	Bacteriophage displaying neutrophil-binding peptide (FGP phage) stimulates neutrophil chemotaxis [76].
**Phagocytosis of viral particles**	Virions are phagocytosed by both neutrophils and monocytes e.g., Herpes virus is phagocytosed, and its envelope proteins are degraded [77]. Monocytes are faster at viral DNA degradation/digestion, neutrophils are more effective in phagocytosis.	Phage particles are phagocytosed by neutrophils, monocytes and dendritic cells [22,23,24,25,26,27].
**Influence on phagocytosis**	Active Mumps and the influenza viruses, inhibited the process of B. anthracis phagocytosis [78]. EBV reduced phagocytic activity of human monocytes, Influenza virus decreased phagocytosis of bacteria by human neutrophils [79,80] and murine macrophages [81] In HIV-positive patients the process of formation of the phagosome was impaired Coxsackie virus exerted an inhibitory effect on the ability of leukocytes to phagocytose, and it was dependent on the time of leukocytes’ exposure to the virus [82] Human neutrophils infected with HCMV increased expression of CD11 receptor (responsible for adhesion to the surface of vascular endothelial cells, migration, and phagocytosis of the particles opsonized with complement), resulting in increased phagocytosis [72]. Japanese encephalitis virus caused a reduction in phagocytosis of red dye by human neutrophils, there was no weakening of phagocytic activity for monocytes [83]. The Newcastle disease virus inhibited the phagocytosis of particles of red oil by: human neutrophils after stimulation with zymosan by and oxygen consumption after the stimulation of the cells with PMA, the activity of the membrane-bound enzyme NADPH was also decreased [84].	There was a weakening in phagocytosis of *S. aureus* by patients’ neutrophils which showed an initial decrease in the process [33].
**Virions’ inactivation**	Viral particles are absorbed into the interior of the phagosome, wherein the lysis takes place (e.g., inactivation of influenza virus by human neutrophils) [79].	It occurs in macrophages [22] and stimulated neutrophils (e.g., phage λ) [85].
**Intracellular killing**	Weakening in bactericidal activity of macrophages and neutrophil functions [80,81].	Lack of stimulation; lack of inhibition in vitro [35] and ex vivo [34].
**Neutrophil degranulation**	Respiratory syncytial virus causes neutrophil degranulation [86].	Neither the purified A3/R phage nor its lysate stimulates neutrophil degranulation [47].
**Production of ROS**	Sendai virus, influenza virus, cytomegalovirus, HIV caused the increased production of ROS by phagocytes (murine splenocytes, human neutrophils, monocytes) [69,70,71]. Sendai virus inactivated by ultraviolet light. Heat-inactivated virus did not stimulate the production of ROS. Only active viral particles stimulate the synthesis of ROS by phagocytes [70]. Weakened production by neutrophils and monocytes (HIV) [87].	Lowered production or lack of stimulation (e.g., T4, F-8, A3/R) [47].
**Adsorption to phagocytes**	Rotavirus, enter the host cells by using the interaction between the viral protein VP4 and αVβ3-integrin on the cell surface [88].	β-integrin [17].
**Influence on apoptosis**	Delay in apoptosis of human neutrophils infected with HCMV [83]. Acceleration of neutrophil apoptosis in HIV patients) [89].	No data.

TNF, Tumor necrosis factor; NFκB, Nuclear factor κB; JEV, Japanese encephalitis virus; EBV, Epstein-Barr Virus; HIV, Human Immunodeficiency virus; HCMV, Human Cytomegalovirus; PMA, Phorbol 12-myristate 13-acetate.
